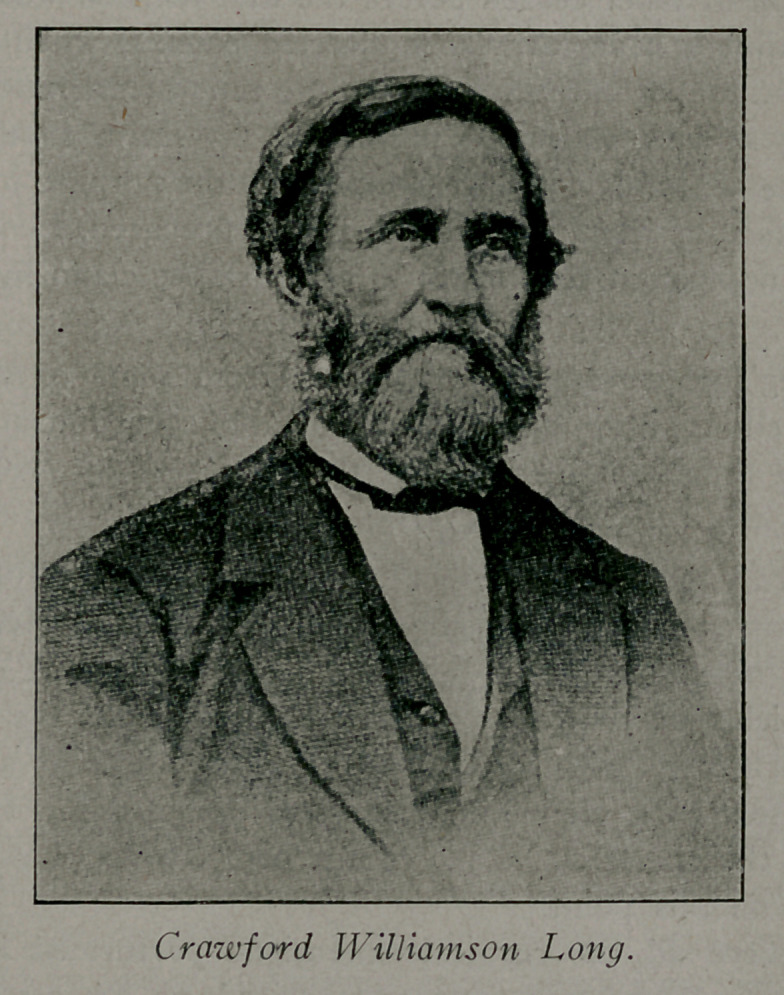# Selections and Abstracts

**Published:** 1912-04

**Authors:** 


### CRAWFORD WILLIAMSON LONG, THE PIONEER OF ANAESTHESIA.

By Dudley W. Buxton, M. D., B. S., M. R. C. P.

*Anaesthetist to University College Hospital and Lecturer on Anaesthetics in University College Hospital Medical School.*

Crawford Williamson Long was the pioneer of anaesthesia and the first to suggest and employ ether inhalation during surgical operations. Although the discovery of anaesthetics has bestowed the greatest blessing upon suffering humanity, its birth has been marked by much polemic and in some cases with scant recognition of those who have done so much for mankind. While disputants claiming for themselves, or their friends, the plaudits due to great discoverers have rent the welkin with their angry cries and clamoured for a material solatium, one man, Crawford Williamson Long, reputed to be actually the first to produce artificial anaesthesia by inhalation of ether, was content to stand aside, calm and dignified, as one who strove to benefit his race while seeking for himself neither guerdon or worldly honor. I propose to submit to your consideration the facts which have been accumulated and which, to my mind, prove Long's right to the world's acknowledgement that he was the pioneer of anaesthesia.

*Views on Possiblity of Anaesthesia at the Time.*

It is impossible to do justice to the man without making at least an attempt to understand the environment in which he worked, and to analyze the professional and public opinion at the time he lived concerning the possibility of anaesthesia. Even so late as 1846 Sir Benjamin Brodie wrote: "Physicians and surgeons have been looking in vain, from the days of Hippocrates down to the present time, for the means of allaying or preventing bodily pain." In his mind were, no doubt, the spongia somnifera of Theodoricus of Lucca, opium, cannabis indica, mandragora, and the whole gamut of nostrums counted through the centuries; the claims of Denis Papin, and of Cardan, whose magnet he averred could abrogate pain; the compression of the carotid arteries by Valverdi; nerve compression, suggested by Ambroise Pare and practiced by James Moore, and subsequently by John Hunter in St. George's Hospital; Wardrop's (1832) bleeding to syncope, adopted by Richerand; and Mesmer's claims to disassociate the astral from the corporeal body. Many methods had been called, but, as the sequel will show, few had been chosen.

*Some Biographical Details.*

But what of the man himself? Crawford Williamson Long was born on Nov. 3rd, 1815, at Danielsberg, Georgia. His people were of high intellectual and social standing; his grandfather, Captain Samuel Long, who had emigrated from Ireland, was a philanthropist and man of affairs, prominent in the war of his troublous times. Captain Long's wife was a Miss Williamson, of Ulster, and her maiden name was perpetuated in that of her grandson. Of Long's father we learn that he was high in the esteem of his fellow-countrymen and the intimate friend of W. H. Crawford, after whom he named the subject of this history. Crawford held important official posts, twice represented his country in France, and was a Secretary of State. Long's mother, although an invalid, by her literary gifts added culture to her home. Thus was Crawford Long brought up amidst refined and public-spirited persons, an environment which could but make for those traits of character which in later life developed him into the best type of the man and of the physician.

A few words will suffice to tell the subsequent story. "Studious and wise beyond his years," Long, whose extreme youth when he went to college earned him the *sobriquet* of "The Baby," graduated brilliantly at Franklin College—now the University of Georgia—when but 19 years old. His chief friend and classmate was Alexander H. Stephens, later to be elected Vice-President of the Southern Confederacy, and to him and Long have been erected monuments in Statuary Hall, Washington, the twain chosen by Georgia as the greatest men of her State. Thence he proceeded to the University of Pennsylvania, and at the age of 23 graduated in medicine. As was customary for those who could afford it, Long spent some time after this in walking the hospitals of New York. The suggestion made by him by his teachers, who recognized his peculiar merit as a surgeon, that he should attach himself to the medical service of the United States Navy, was discountenanced by Long's father, and at the latter's instance he settled as a general practitioner in Jefferson, Georgia, and commenced civil practice in 1841 at the age of 26.

*Operations on Hypnotized Patients.*

It should be remembreed that it was in this same year that Braid, of Manchester, made his trial of the "neurhypnotic trance," and Esdaile in India sucessfully operated upon hypnotized patients. Elliotson, who had migrated from St. Thomas's to University College Hospital, was then giving the full weight of his great mental power to mesmerism, although his book on "Surgical Operations Performed in the Mesmeric State without Pain" was not published until 1843. There is no doubt that at the time of which we write mesmerism, or the hypnotic trance, was regarded as the accomplished fact of anaesthesia, and that in the United States many of the leading men in medicine and surbery accepted it as the long-hoped-for panacea whereby suffering humanity could pass unflinchingly through the ordeal of the surgeon's knife. In France, Richerand had tried it and pronounced for its value, and other surgeons scarcely less eminent were willing to swallow the doubtful reputation of Anton Mesmer so long as they could benefit their patients by employing methods which had been exploited by his fertile brain.

Inhalational anaesthesia thus started with a heavy handicap. In 1846, to anticipate, the editors of the New Orleans Medical and Surgical Journal wrote, *apropos* of ether inhalations: "That the leading surgeons of Boston could be captivated by such an invention as this under such auspices and upon such evidences of utility and safety as are presented by Dr. Bigelow excites our amazement. Why, mesmerism, which is repudiated by the *savants* of Boston, has done a thousand times greater wonders and without any of the dangers here threatened. What shall we see next?" *En passant*, let it be remarked that the sapient editors were condemned to see many things apparently unpalatable. But putting this aside, the quotation appears to show that the utility of mesmerism was widely accepted and constantly practiced during surgical operation, even if the Bostonian *savants* condemned it; and further, that any fresh departure in the direction of promoting painless surgery was open to the most embittered criticism. The importance of this fact will be made manifest in the sequel of our narrative.

*Knowledge then Current about Ether and Nitrous Oxide Gas.*

We must, however, retrace our steps for a brief space and try to obtain a clear idea of what was the current knowledge about ether before Long's first trials of its powers as an anaesthetic. In the books dealing with drugs and poisons ether found a place. It had been known since the thirteenth century and although some of its merits were recognized it was regarded as so dangerous as to be taboo. However, when, through the enthusiasm of Dr. Beddoes, the work of Priestly, and the discovery of oxygen and nitrous oxide, pneumatic medicine became a vogue ether again assumed prominence.

The foundation of the Hotwells Hospital at Clifton, Bristol, by Dr. Beddoes gave that astute physician a wide experience in inhalational medication. He had studied Mayou's experiments and was familiar with Priestley's work, so that when young Humphrey Davy, freed from his indentures to a doctor in Penzance, became his assistant, Beddoes was able to study the gases, the effects of which in the treatment of disease he has given in his delightful collected writings in four volumes. Of the volume entitled "Considerations on the Medicinal Use and on the Production of Factitious Airs," the first part was Beddoe's work, the second being due to James Watt, the engineer. In one of these volumes we find a letter from Dr. Pearson, of Birmingham, the accepted authority in his day upon therapeusis both in this country and in the States. In it he says that Beddoe's researches on "Factitious Airs" had led him to try "the vapour of ether" to relieve the suffering of phthisical patients and with benefit to them. Further, when Mitchell's book on chemistry appeared Beddoes found to his chagrin that the chemist declared nitrous oxide to be a "virulent poison," so he recommenced experiments with oxygen and nitrogen as well as with nitrous oxide gas. Davy, who undertook these researches, came to the conclusion that Mitchell was wrong, and that "the gaseous oxide of azote is perfectly expirable (*sic.*)" He also announced that its inhalation cured the pain of an aching tooth, and added: "It may probably be used with advantage during surgical operations in which no great effusion of blood takes place." Alas, no one took the hint, and nitrous oxide remained for nearly 50 years the chief stock-in-trade of traveling lecturers, who gave the gas to members of their audience to provoke exhilaration and semi-unconscious gyrations. These "frolics" were well known, and M. Filvee, in his "Lettres sur l'Angleterre" (1802), mentions these revels as one of the many follies to which English people were prone.

These practices became also common in the States, and it will be remembered it was at one of them that Horace Wells was present when he conceived the idea of using the gas as an anaesthetic. This, of course, happened much later, in 1844. But nitrous oxide gas needs a plant for its accurate manufacture, so that Pearson's suggestion of employing ether vapour as an exhilarant found ready acceptance. Cullen, whose works were read widely both here and in America, advocated ether, and Warren, of Boston, extolled its use in place of nitrous oxide. We see then that in 1841, when Long commenced practice, it was common knowledge that nitrous oxide produced exhilaration, but the suggestion of its employment as an anaesthetic by Davy was forgotten; ether was recognized as producing similar effects, but if we except the doubtful hint in the Journal of Sciences and Arts associated with the name of Farady (1818), no one had grasped its greater merits, while the books of Pereira and others cautioned against its employment, since stupor and death they averred might readily be brought about. Long, it appears, had when a student actually inhaled ether during an "ether frolic,'" and was so far familiar with its effects.

*Long's Early Experiments.*

Jefferson in those days was an isolated country village—

one might almost say, "the world forgetting, by the world forgot"—so that Long had to pursue his own way relying upon himself and practically out of touch with the centres of surgical thought. His personal charm and high scientific attainments made his house a place of social resort for the neighborhood. Let Long himself tell us of the fateful happenings of December, 1841. He says:

In the month of December, 1841, or January, 1842, the subject of the inhalation of nitrous oxide gas as introduced in a company of young men in this village (Jefferson), and several persons present desired me to produce some for their use. I informed them that I had no apparatus for preparing or preserving the gas, but that I had a medicine (sulphuric ether) which would produce equally exhilarating effects; that I had inhaled it myself, and considered it as safe as the nitrous oxide gas. One of the company stated that he had inhaled ether while at school, and was then willing to inhale it. The company were all anxious to witness its effects. The ether was introduced. I gave it first to the gentleman who had previously inhaled it, then inhaled it myself, and afterwards gave it to all persons present. They were so much pleased with the exhilarating effects of ether, that they afterwards inhaled it frequently, and induced others to do so, and its inhalation soon became fashionable in this country, and in fact extended from this place through several counties in this part of Georgia.

On numerous occasions I have inhaled ether for its exhilarating properties, and would frequently, at some short time subsequent to its inhalation, discover bruises or painful spots on my persons, which I had no recollection of causing, and which I felt satisfied were received while under the influences of ether. I noticed my friends while etherized, received falls and bangs, which I believed were sufficient to produce pain on a person not in a state of anaesthesia, and on questioning them they uniformly assured me that they did not feel the least pain from these accidents. These facts are mentioned that the reasons may be apparent why I was induced to make an experiment in etherization.

*First Operation under Ether Anaesthesia.*

The first trial of his theory was made on March 30th, 1824, and the narrative is given in Long's published account of his experiments, James M. Venable was etherized by Long, who poured ether upon a towel, and when the patient was profoundly unconscious he removed a cyst from the back of the patient's neck. No pain was felt. This was four and a half years before Morton's first case. Subsequently Long used ether on several occasions, eight it is said, and every case was successful. That he did not employ it more largely was due, firstly, to the fact that his practice was one in which operations were seldom demanded, and secondly, because the older practitioners around him urged upon him the "danger" of the method and the disastrous results which would follow a fatal accident, such an accident as they were good enough to say must soon occur. Indeed, Long was more than once threatened with pains and penalties by the community in which he lived if he persisted in his ether practices!

*Reason of Long's Silence.*

Now, although Long did not conceal his work, for it was known widely in the neighborhood, as is shown by the facsimile letter which is here reproduced (Fig. 3), he took no occasion until later to read a paper before a medical society dealing with his experiments. This silence has been misconstrued. The simplest justice, however, must explain Long's reticence in a sense which redounds wholly to his credit. Long believed in his method, but recognized that it needed careful working out; he patiently experimented when patients seemed suitable; when they were not available he tried it on himself and on his pupils. knew that the public were in favor of mesmerism, a system which he regarded with disfavor and he had no surgeon of eminence in his neighborhood to whom he could apply for extended experience. The doctors who knew of his efforts were averse to them, and so he preferred to wait and gain experience before attempting to exploit a discovery which might have less in it than appeared at first sight. He was alive to the fact that some might believe they found in his practice a development of hypnotism, an anaesthesia by suggestion. But while he waited events were happening in the bigger world of which Long recked nothing.

Jefferson, Feby. 1st, 1842.

*Copy of Facsimile Letter.*

Dear Bob:

I am under the necessity of troubling you a little. I am entirely out of Ether and wish some by tomorrow night if it is possible to receive it by that time. We have some girls in Jefferson who are anxious to see it taken.

Your friend,

C. W. Long.

This letter written to me by Dr. C. W. Long in which he ordered the Ether that he performed the first surgical operation on a patient under the influence of that drug—a wart was removed from the neck of a young man—Mr. James Venable, without giving him any pain. It was a complete success. This statement is true as I learned it from Dr. C. W. Long.

R. H. Goodman.

A copy of the facsimile letter written in 1842 to Mr. R. H. Goodman, and a note written upon the enfold of the letter by the hand of R. H. Goodman. The ether was used for an "ether frolic" and for the first operation upon J. M. Venable.

GEORGIA, FULTON COUNTY:—

I, Ange Delaperriere, M. D., do certify that I resided in Jefferson Jackson County, Georgia, in the year 1842 and that sometime in that year I heard James M. Venable, then of said State and County now deceased, speak of Dr. C. W. Long, then of Jefferson in the County of Jackson, Georgia, now of Athens, Georgia cutting two tumors from his neck while under the influence of Ether without pain or being conscious of the performance of the operations.

I do further certify that the fact of Dr. C. W. Long using Sulphuric Ether by inhalation to prevent pain in surgical operations was frequently spoken of and notorious in the County of Jackson, Sate of Georgia, in the year 1842.

I do further certify that the said James M. Venable was born and raised near Jefferson and was regarded as a young man of truth and veracity.

A. DeLaperriere, M. D.

Sworn to and subscribed before me this 30th day of March, 1854.

N. H. Pendergrass, J. P.

### *Morton and "Letheon."*

Horace Wells had "discovered" nitrous oxide gas, had tried it and had been successful in Hartford, Connecticut, 1844. His essay before the surgical world in the General Hospital, Massachusetts, had been a fiasco; he (Wells) had been shrieked out of the theatre with cries of "humbug." With or without the aid of Dr. Jackson, a man of scientific attainments and State geologist and chemist, Morton had administered ether vapor to himself, subsequently to one or two patients, and ultimately had been permitted by Warren, the surgeon, to admnister his nostrum to a patient in the "General Hospital." "This, gentlemen," Warren had said, "is no humbug:" anaesthesia by inhalation was an accepted fact.

These events occurred in 1846, but sadly enough the "ether controversy," which embroiled Morton, Jackson and Wells, involves much that we would rather not dwell upon, since the details are far from pleasant reading. We must, however, refer to some points since they connect the story with that of Crawford Long. We spoke of Morton using a "nostrum;" it was sulphuric ether with flavoring additions, and was termed "Letheon." Its identity with ether was challenged, and eventually the medical profession refused to allow further public trials without a disclosure of its nature and composition. The attempt was made to protect letheon by a patent, and to restrict its employment unless a royalty was paid. An English patent was actually granted. W. T. G. Morton was not a medical man, and probably saw no objection to reaping a golden harvest from what he no doubt honestly believed to be his discovery. This restriction on the use of letheon has been denied, but the letter of Dr. Charles A. Davis appears to substantiate the fact.*

*Letter from Dr. Davis written 12 years after the patent was granted to Morton for his letheon, showing that he attempted to enforce patent rights: "U. S. Marine Hospital, Chelsea, Massachusetts, April 1859. Dr. Crawford W. Long, Athens, Georgia. Sir,—Hon. Judge Hyllier, Solicitor of Treasury Department, informed me about a year since, and recently repeated the same, that some years since you used sulphuric ether as an anaesthetic and had a record of the same. If it is not asking too much of you I would be grealy obliged if at your earliest convenience you would forward me a statement of the fact. I take the liberty to ask this of you because Mr. W. T. G. Morton, to whom in conjunction with Dr. C. T. Jackson a patent was granted in November, 1846, for using ether, has brought a suit against me as a Government Officer for an infringement of his patent. Judge Hillyer was confident that you could furnish me with proof sufficient to satisfy a jury that you used it in this way before he or Jackson claimed to have made the discovery. I should have asked for these proofs through my attorney and had them properly witnessed, etc., but the Secretary of the Treasury having decided that I used the article on my own responsibility, and therefore the Government were not bound to defend me, I wish to save as much expense as possible.—Very respectfully (signed) CHARLES A. DAVIS, M. D., Physician and Superintendent.' '

We know the sequel; the world awoke to the anaesthetic value of ether. There can be no question that whatever motives actuated him, Morton's public exhibitions of ether in an important surgical centre were the direct means of publishing the discovery to all civilized communities. Failing to support the rights which he fondly hoped would protect his patent of letheon, Morton sought to secure a grant from Congress, and to be proclaimed the discoverer of ether, and the first to apply it for the purposes of anaesthesia. Dr. Jackson advanced that he had a prior right, asserting that he had suggested the use of ether to Morton, and that the latter's employment of it was at his (Jackson's) instance. Into the merits of this controversy we need not enter, since both Morton and Jackson had been anticipated by Long.

The reasons why Long did not publish his first cases have been pointed out already. However, when Morton's success was bruited about, the duty of making a statement concerning his own cases was brought home to Long, and he put himself into communication with the editor of the Southern Medical and Surgical Journal. In 1849 we find in the columns of that journal the following: "A few months ago Dr. Long informed us of his early attempts at etherization in surgery. He was then informed that any claims set up at this late day to priority would be severely criticized, if not violently resisted; and that he had best, therefore, do all he could to fortify his position."

Long, in his communication to the Medical Society of Georgia (December, 1849,) explains that so soon as he saw from the current medical literature (1846) that letheon had been successfully used, he actually commenced a paper for publication detailing his own experiences with sulphuric ether. Unhappily, the exigencies of his practice intervened and the paper was never completed.

*The Rival Claims.*

In 1849 Morton petitioned Congress for a monetary reward for his discovery, and his claim was opposed by Jackson and Wells. In 1854, after much persuasion, Long was induced to write to Senator Dawson detailing his own work. The Senator sent Dr. Jackson a curious choice when it is remembered that that gentleman was one of those who claimed the laurels of having discovered ether. The interview took place in Athens, Georgia, to which town Dr. Long had removed in 1851, and Long told his story simply but so effectively as to convince Jackson of its truth.* The matter was ventilated in Congress by Senator Dawson, and although Morton's petition was never granted, yet to Crawford Long no recognition was vouchsafed. It is significant that Dr. Jackson urged upon Long that they, Jackson and Long, should unite interests, and as partners claim that they had respectively discovered the use of ether as an anaesthetic, and actually essayed experiments and successfully proved the safety and value of the method. Long was not tempted; he declined in words of striking dignity. He writes: "Our claims are rival, and permit me, sir, to say that although our claims are conflicting, I would not knowingly say anything in the article (he was preparing a full satement of his work in the form of an article) which would be displeasing to you. ------------Still, it becomes each one of us to use all honorable means to advance his own claims, and I know you will not blame me for attending to this matter, which so much concerns my reputation." So the matter dropped, and men forgot Long or minimized his work, and on Ether Day celebrations to Morton is accorded the palm. In 1877 Dr. Marion Sims championed Long's cause, but, unfortunately, his communication so well conceived was hurriedly executed and introduced the inaccurate statement that S. C. Wilhite had suggested to Long the idea of using ether. Wilhite himself contradicted this, and his letter

---

Dr. Jackson published a full account of this interview and the steps he took to verify Dr. Long's statements. See Boston Medical and Surgical Journal, April 11th, 1861.

is extant. As a matter of fact, he did not come to live with Long as a student until 1844.

*Close of Story of Long's Life.*

So we come to the close of the story of Long and his great discovery, the story of a simple-minded man who, amidst the arduous labors of a scattered country practice, conceived a great idea and, in the teeth of opposition, and though haunted by the fear lest disaster might arise and ruin his professional name and reputation, yet had the courage to test his theory by experiment and so obtained proof of its accuracy. Surely such a man was worthy to be the author of so momentous a discovery, and of being called one of the greatest men brought forth by a mighty nation.

A few words will suffice to trace the closing years of Long's useful life. He took no further steps to enforce his claims but patiently labored for his country and his people through the troublous years of the war, and at length, when brighter days returned, he built up again his practice and ultimately died in harness, his last words being an anxious inquiry as to the welfare of a patient at whose bedside he was when his mortal sickness seized him.

There have been memorials to the memory of Long, the discoverer of ether anaesthesia, one in Paris and one at Jefferson, Georgia, and in Boston men see a monument in white marble bearing the inscription: "To the Discoverer of Anaesthesia." It bears no name.

Shall we not in England accord the first place to Long, and in no unfriendly spirit claim for Morton and Wells less exalted niches in the Temple of Fame; for we may not forget that to them, and indeed to Dr. Jackson, the world owes very much, since, if not the first to employ ether as an anaesthetic, they made themselves the heralds of an epoch-making discovery, and they were in ignorance when they did so that Crawford Williamson Long had anticipated them?

*Statement of Dr. Long.*

The following statement made by Dr. C. W. Long in 1849

to the Medical Society of Georgia* is of great interest:—

For nearly three years the various medical journals have contained numberous articles on the employment of sulphuric ether by inhalation, for the purpose of rendering patients insensible to pain during surgical operations.

Atlanta, DeKalb Co., Ga. Apr. 3rd 1853.

C. W. Long, M. D.:—

It affords me pleasure to certify and I do hereby affirm that I saw you perform an operation upon Mr. James M. Venable, to wit, the cutting out and removing of a tumor from the neck of the said James M. Venable.

The operation was performed when Mr. Venable was under the *influence of sulphuric ether,* produced by inhaling the same. I was intimate with Mr. Venable at the time of the operation, and afterwards frequently conversed with him upon the subject and he often told me that the operation produced no pain. The operation was performed in the town of Jefferson, Jackson County, and State of Georgia in the year *One Thousand Eight Hundred and Forty-Two.*

Yours, etc.,

Wm. H. Thurmond.

The first notice I saw of the use of ether, or rather of Dr. Morton's "Letheon," as an anaesthetic, was in the editorial of the Medical Examiner for December, 1846, in which the editor gives the following extract from a paper by Dr. H. J. Bigelow, contained in the Boston Journal. "The preparation (Letheon) is inhaled from a small two-necked glass bottle, and small two-necked glass globe, and smells of ether, and is, we have little doubt, an ethereal solution of some narcotic substance."

Having on several occasions used ether since March, 1842, to prevent pain in surgical operations, immediately after reading this notice of "Letheon" I commenced a communication to the editor of the Medical Examiner for publication in that journal to notify the medical profession that sulphuric ether when inhaled would of itself render surgical operations painless, and that it had been used by me for that purpose for more than four years.

I was interrupted when I had written but a few lines and was prevented by a very laborious country practice from resuming my communication until the Medical Examiner for January, 1847, was received which reached me in a few days after reading the December number. It contained several articles, giving accounts of different experiments in etherization, in which surgical operations were performed without pain. On reading these articles I determined to wait for a few months before publishing an account of my discovery and see whether any surgeon would present a claim to having used ether by inhalation in surgical operations prior to the time it was used by me.

A controversy soon ensued between Messrs. Jackson, Morton and Wells, in regard to who was entitled to the honor of being the discoverer of the anaesthetic powers of ether, and a considerable time elapsed before I was able to ascertain the exact period when the first operations were performed. Ascertaining this fact, through negligence I have now permitted a much longer time to elapse than I designed, or than my professional friends with whom I had consulted advised; but as no account has been published (so far as I have been able to ascertain) of their inhalation of ether being used to prevent pain in surgical operations as early as March, 1842, my friends think I would be doing myself injustice not to notify my brethren of the medical profession of my priority of the use of ether by inhalation in surgical practice.

I know that my interests have suffered from not making an earlier publication, and I would not be persuaded at this late stage of the ether controversy to present my claim to being the first to use ether as an anaesthetic in surgical operations, if I were not fully satisfied of my ability to establish its justness.

*Account of First Two Operations.*

Then follows Dr. Long's statement setting forth the chain of reasoning which led him to employ ether as an anaesthetic. We have already quoted this. Long continues:—

The first patient to whom I administered ether in a surgical operation was Mr. James M. Venable, who then resided within two miles of Jefferson, and at present lives in Cobb County, Georgia. Mr. Venable consulted me on several occasions in regard to the propriety of removing two small tumors on the back of his neck, but would postpone from time to time having the operations performed from dread of pain. At length I mentioned to him the fact of my having received bruises while under the influence of the vapor of ether without suffering, and as I knew him to be fond of and accustomed to inhale ether, I suggested to him the probabiliy that the operations might be performed without pain, and proposed operating on him while under its influence. He consented to have one tumor removed, and the operation was performed the same evening. The ether was

*Crawford Williamson Long.*

given to Mr. Venable on a towel, and when fully under its influences I extirpated the tumor. It was encysted and about half an inch in diameter. The patient continued to inhale ether during the time of the operation, and when informed it was over seemed incredulous until the tumor was shown him. He gave no evidence of suffering during the operation, and assured me after it was over that he did not experience the slightest degree of pain from its performance. The operation was performed on March 30h, 1842.

The second operation I performed upon a patient etherized was on June 6th, 1842, and was on the same person for the removal of another small tumor. This operation required more time than the first, from the case of the tumor having formed adhesions to the surrounding parts. The patient was insensible to pain during the operation, until the last attachment of the cyst was separated, when he exhibted signs of slight suffering, but asserted after the operation was over that the sensation of pain was so slight as scarcely to be perceived. In this operation the inhalation of ether ceased before the first incision was made. Since that time I have invariably directed paients, when practicable, to continue its inhalation during the time of operation.

Having so long neglected presenting my claim to the discovery of the anaesthetic power of ether, for the purpose of satisfying the minds of all of its justness, I have procured, I conceive, a sufficient number of certificates to establish the claim indisputably.

In Long's paper appear certificates from some of his patients as well as from eye-winesses of the operation. Fig. 4 is a copy of a fac-simile of one of these.

*Other Operations.*

My third experiment in etherization was made on July 3rd, 1842, and was on a negro boy, the property of Mrs. S. Hemphill, who resides nine miles from Jefferson. The boy had a disease of a toe, which rendered its amputation necessary, and the operation was performed without the boy evincing the least sign of pain. I present Mrs. Hemphill's statement of the report the boy gave her of the operation on his return home, which I conceive is sufficient on this point.

These were all the surgical operations performed by me during the year 1842, upon patients etherized, no other case occurring in which I believed the inhalation of ether applicable. Since 1842 I have performed one or more surgical operations annually on patients in a state of etherization.

The question will no doubt occur, Why did I not publish the results of my experiments in etherization soon after they were made? I was anxious, before making my publication, to try etherization in a sufficient number of cases to satisfy my mind that anaesthesia was produced by the ether, and was not the effect of the imagination or owing to any susceptibility to pain in the persons experimented upon.

At the time I was experimenting with ether there were physicians high in authority and of justly distinguished chhracter, who were advocates of mesmerism, and recommended the induction of the mesmeric state as adequate to prevent pain in surgical operations. Notwithstanding thus sanctioned, I was an unbeliever in the science, and of the opinion that if the mesmeric state could be produced at all it was only on those of strong imaginations and weak minds," and was to be ascribed solely to the workings of the patients' imaginations. Entertaining this opinion, I was the more particular in my experiments in etherization.

Surgical operations are not of frequent occurrence in a country practice, and especially in the practice of a young physician, yet I was fortunate enough to meet with two cases in which I could satisfactorily test the anaesthetic powers of each. From one of these patiens I removed three tumors the same day. The inhalation of ether was used only in the second operation, and was effectual in preventing pain, while the patient suffered severely from the extirpation of the other tumors. In the other case I amputated two fingers of a negro boy. The boy was etherized during one amputation and not during the other; he suffered during one operation and was insensible during the other.

I have procured the certificates of the lady from whom the tumors were removed and of her husband, who was present and witnessed the operations. These certificates were produced in preference to those establishng other operations, because they not only show that the experiments were continued from year to year, but also show that they were conducted so as to test the power of etherization.

After fully satisfying myself of the power of ether to produce anaesthesia, I was desirous of administering it in a severer surgical operation than any I had performed. In my practice, prior to the published account of the use of ether as an anaesthetic, I had no opportunity of experimenting with it in a severer surgical operation, my cases being confined, with one exception, to the extirpation of small tumors and the amputation of fingers and toes.

I have stated that ether was frequently inhaled in this and some of the adjoining counties for its exhilarating effects, and although I am conscious that I do not deserve any credit for inroducing is use for that purpose, yet as others through their friends have claimed to be the first to show it safety, most of the certificates I have obtained establish the fact of its frequent inhalation for is exhilarating effects. I met with R. H. Goodman, who was present the night ether was first inhaled in Jefferson, and who removed to Athens, and introduced its inhalation in that place, and presented his certificate. All the young gentlemen who were present the night I first administered ether, with one exception, are living, and their certificates can be produced, if necessary.

I have now, in a very concise manner, presented a "plain unvarnished" account of some of my experiments in etherization, and have said nothing of the comparative methods of ether, and other anaesthetics, because that was foreign to my present subject. Had I been engaged in the practice of my profession in a city where surgical operations were performed daily, the discovery would, no doubt, have been confided to others, who would have assisted in the experiments, but occupying a different position, I acted differently, whether justifiable or not. The result of my second experiment in etherization was such as to lead me to believe that the anaesthetic state was of such short duration that ether could only be applicable in cases in which its effects could be kept up, by constant inhalation, during the time of the performance of the operation. Under this impression, up to January, 1847, I had not used ether, but in one case, in extracting teeth, and thus deprived myself of experimenting in the only class of cases which are of frequent occurrencee in a country practice.

While cautiously experimenting with ether, as cases occurred, with a view of fully testing its anaesthetic powers, and its applicability to severe as well as minor surgical operations, others more favorably situated engaged in similar experiments, and consequently the publication of etherization did not "bide my time." This being the case, I leave it with an enlightened medical profession to say whether or not my claim to the discovery of etherization is forfeited, by not being presented earlier, and with the decision which may be made I shall be content.

The fac-simile of the original entry in Dr. Long's account-book of his charge made to the first patient, J. M. Venable, possesses interest (see Fig. 5).

JAMES VENABLE
To Dr. C. W. Long, Dr.

1842
January 28th, sulphuric ether .... .25
March 30, Ether and extracting tumor .... 2.00
May 13, Sulphuric ether .... .25
June 6—Extracting tumor .... 2.00

GEORGIA, JACKSON COUNTY:

I. P. F. Hinton, clerk of the Superior Court of said county do certify that the above account is a correct copy of an original entry made in his book for medical services for the year 1842.

Given under my hand and seal of this office this 27th of March, 1851.

P. F. HINTON, Clerk Superior Court.

*Ether*, 1842-1911.

The narrative given by Long of his first administration of ether to a patient in 1842, that by Morton, which is more detailed, referring to his demonstration of the effects of Letheon on a patient in the Massachusetts General Hospital (1846), and that given in great detail of the first ether operation performed in a London hospital—viz., University College Hospital—when Liston operated, reveal the fact that very little was known about anaesthetics and less about methods. In the one case a towel was employed, in the other two a primitive inhaler, consisting of an ether chamber and a series of tubes connecting it with a face-piece.

After Simpson introduced chloroform at the end of 1847, ether, at all events in this country, was neglected in favor of the newer claimant, chloroform. The perils of the latter incident to the methods adopted in its exhibition soon led to fresh attempts being made to employ ether or some mixture of it and chloroform. The committee of the Royal Medical and Chirurgical Society in its report published in 1864 extolled ether's safety, but pointed out as its inherent drawback that the induction of anaesthesia by it was too slow for convenience. Then came the rational attempts of Clover, Ormsby, and many more to remove this disability by the use of closed inhalers. No practical attempt was, however, made to study a percentage method for etherization. The very safety of the drug became its chiefest danger, since etherists were so obsessed by the fact that ether does not lower blood pressure or cause cardiac collapse through depression, that they failed to recognize the perils incident to over-stimulaiton, especially in ashenic persons. The dangers of post-operative chest troubles were not existent in the pioneer days of ether because the operations performed were comparatively brief and the surgeons taught in pre-anaesthetic days prided themselves on their celerity in operating, and, indeed, were appraised by the public for this quality. To-day there is no haste, the advance of surgery has invaded the regions once immune from the knife; if ether is adopted for this wider range of operations it is necessary that methods of using it must follow on other than the traditional lines. To safeguard against excessive dosage we rely on mixed methods, such as the preliminary hypodermic injection of scopolamine, morphine, and atropine; we adopt an open mask, evaporating from an enormously expanded area provided by many folds of gauze, and so obtain a more complete nebulation of our vapor; we introduce ether directly into the blood-stream in an artificial circulating fluid of physiological saline solution by intravenous infusion, with the hope of maintaining an equable and low-grade partial saturation of the neural tissue. In every case, we must remark, the supreme difference consists in the fact that we have replaced a method of excessive dosing by one of moderation and in most instances capable of rapid vibration in the strength of ether employed. We have been too overborne by *a priori* reasoning, too obedient to traditional authority. Whether our newer methods may not introduce fresh dangers, we can not as yet say; if they do it will probably be because our technique is at fault, and this must be amended. It is startling when we think of the early workers to find the modern etherist safely and easily encompass anaeshesia with ether for tongue or jaw operations. Yet such is the case. By the intratracheal insulation method now so efficiently carried out in America, we find ether conveyed into the lungs without the inconveniences formerly incident to the method of introducing ether by oral inspiration. The experience gained gives promise of even more efficient plans of using ether, of saving life, and enabling the modern surgeon to perform his tasks, often almost daunting in their complexity and difficulty, without the added anxiety of an anaesthesia either imperiling the patients life or necessarily imperfect owing to the patient's reaction towards the drug employed. If Long's work was the first step towards what we have achieved to-day, and it was so, to him we owe this much, that we do his memory great and abiding honor. But we must realize, also, that anaesthesia to-day is on its trial, it must advance and trample on tradition and rely upon experiment unless we are content to forsake the hope of founding a science, and are willing to content ourselves with a mere handicraftsman's place in the ranks of the medical profession.

I desire to express my thanks to Mrs. Long Taylor, through whose kindness I have been furnished with documentary evidence of the accuracy of the facts I have advanced about her father, Crawford W. Long; also to Dr. George Foy, of Dublin, to whose unique knowledge of this matter and collection of memorials of Crawford Long I have been most generously made welcome.

---

### CLINICAL SOCIETY OF THE NEW YORK POLYCLINIC MEDICAL SCHOOL AND HOSPITAL, MEETING OF MARCH 4TH, 1912.

Case of Foreign Body in the Eye; X-ray Localization; General Infection of the Eye Controlled by Urotropin.—Case presented by Dr. Earl Conner.

The patient, a young man of 22, called upon Dr. Conner, with a history of being employed in hammering or using a chisel on a piece of cold steel. A piece of the metal flew and struck him in the eye. When seen four days later, the eye was swollen and painful; the anterior chamber was filled with pus, and on the nasal side of the globe there was a minute punctured wound, about 4 mm. in size. The patient had not slept the night before. He was informed that the eye was infected and that in all probability would be lost, though every effort would be made to save it.

On admission to the Hospital, the test as made with the Giant Magnet. The result, however, was neither positive or negative. If there is a foreign body in the eye it is apt to give pain on being brought into the field of the magnet. In this case, the temporal side of the eye was brought into the field of the magnet, and the patient experienced sharp pain, but after a dozen trials no foreign body could be located at any point. The patient was put to bed, given artopine, and hot applications of calomel.

The pupil was dilaeand and pus in the anterior chamber absorbed, and the interior of the eye examined. A yellow reflection was obtained, which was evidence of infection of the vitreous. The inflammation increase d from day to day, up to the tenth day before it was possible to control it. After the patient was put to bed he was given calomel, followed laer by seven and a half grains of uretropin three times a day for two weeks. This drug has been administered in a large number of cases of eye infection with very favorable results. Dr. Conner said that he had had three cases of eye infection in the past few weeks in which the infection had apparently been controlled by the use of Urotropin.

Skiagraphs had been made of the eye for the purpose of determining the presence or absence of a foreign body. The size of the foreign body as determined by the skiagraph was 1 mm. by 1 1-2 mm.

As a rule hese cases go on to the loss of the eye. An infection of the vitreus is seldom or never arrested. The usual termination is perforation of the eye and hemorrhage. The inflammation in this instance has been controlled, the pain relieved, the patient has perception of light, and there is hope of saving the eye.

Two Cases Presened by Dr. C. G. Child. Operation subsequent to Gillian operation.

Dr. Child reported two cases operated on by the Gillian mehod which had returned after being operated upon. He said hat neither case had been relieved by his method, and were suffering from pelvic pain with dragging on the abdominal wall and this in spite that both operations had been done by most competent men.

Dr. Child said hat he objected to the operaion as he intesines and omentum later became incarceraed in the pelvis. It produced pathological conditions which were worse than the original displacement of the uterus. Varicose conditions followed and an even worse state of things supervened in the formation of pockets in the pelvis.

In both the cases he reported, pockets had formed in one of which the sigmoid was exensively adherent, and constipation had been obstinate.

Dr. Tovey said that Dr. Wells used to o the operation until he found that the patients came back with the uterus in the same position, and all the troubles increased, or else they complained of pain in the side where he ligaments were attached. They had abandoned the operation.

Haematuria in a Multipara: Unknown Cause.—Presented by Dr. Ward B. Hoag.

Dr. Hoag reported a case of haemauria in a multipara forty years of age, in her fourh pregnancy. At six and a half months she developed without any discomfort, a considerable quantity of blood in the urine. Beyond the presence of blood there was no pain or discomfort of any kind.

The patient was put to bed, irrigations of alum solution used, and rest enjoined for ten days. It had no effect on the bleeding. She went on to full terms and was delivered in a perfectly normal way. The haemorrhage continued for two weeks after the child was born, and then stopped as suddenly as it had begun. Dr. Hoag thought it was the result of intra-abdominal pressure, perhaps the rupture of a small blood vessel. The same night there was a little fleshy plug passed in the urine which was the only thing ever seen, and coincident with this the haemorrhage stopped and has not since returned.

Dr. Shears said that although the haemorrhage might be due to toxic conditions, in te present instance there were no signs of a toxemia. Ruling out local papilloma or a ureteritis, he thought that six and a half or seven months was not too early to exert pressure symptoms sufficiently severe to produce haemorrhage. From a careful examination of the bladder and the absence of stone or other aggravating cause he should be inclined to attribute the bleeding to this cause.

### OPENING OF THE NEW DISPENSARY BUILDING OF THE PHILADELPHIA POLYCLINIC AND SCHOOL FOR GRADUATES IN MEDICINE.

The opening of the new dispensary of the Philadelphia Polyclinic and College for graduates in medicine, 18th and Lombard streets, was celebrated on the afternoon of February 5th, 1912, for a formal reception tendered by the President of the Board of Trustees, Mr. Herbert L. Clark, to the Board, the Incorporators of the Hospital and the members of the Medical Staff.

This institution was organized in the year 1882, for the purpose of meeting a long-felt demand for post-graduate teaching in the city of Philadelphia. It was the first institution to devote its labors to this work in Philadelphia, and it continues to be the only institution of this character. In the various years of its existence, it has extended instruction to many students from all parts of the country, as well as from all parts of the world.

Aside from this very important line of work, is has rendered especial service to the city of Philadelphia in that it has conducted one of its largest charitable medical services. From the time of its organization up to the present, there has been an increasing number of patients treated from year to year, until at the close of the present year the official records of the institution show that in the year 1911, in its out-patient service, 17,769 new patients have been treated, with a total number of 78,386 visits; that in its accident emergency ward 8,706 new patients have been treated, with a total of 9,104 visits; an that in the wards of the wards of the hospital there have been treated 1,746; and that 1,167 operations under ether have been performed. This enormous service began to overtax the capaciay of the institution several years ago. Its Board of Trustees, appreciating this fact, in 1907 undertook construction of the building which was formally opened February 5.

This building is constructed especially to accommodate the patients who visit the vispensaries and accident wards. Incidentally, however, it makes available to the institution much needed space in the original building for the increase of its beds for n-patient service, making available space for more satisfactory and complete operatng room for private patients, more comfortable accommodation of its students, a better housing of its executive force, and at the same time, separate entirely the accident ward and out-patient service from the hospital.

The new building has been made possible only through the generous support of public-spirited citizens, the untiring efforts of its Board of Trustees, and generous appropriations of the Legislature of the State of Pennsylvania.

It is located on the property adjoining the older building, with the entrance from Naudain Street. This location is in a portion of the city in which charitable work is greatly demanded, as evidenced by its enormous dispensary service.

The building is constructed on the most approved fire-proof plan, with the latest ventilating devices, with especial view of obtaining the maximum amount of light and air space. The construction is absolutely hygienic, with all the plumbing exposed, all floors made of concrete, all corners and angles rounded, the elimination of all dust-collecting surfaces, with walls and woodwork finished with glazed surfaces.

The furnishings and equipment of the institution are new in their entirety, and have been selected with a special view to ease of cleanliness. Each of the various dispensaries will be equipped with the most approved modern appliances for the treatment of disease of every character, and to each dispensary will be attached a laboratory equipment to aid in the study and diagnosis of disease. The accident emergency suite will be one of the most completely equipped and up-to-date in the city of Philadelphia. An arrangement has been made for the separation of the sexes, and modern bath-room appliances have been provided.

The waiting rooms for the patients are not only commodious, but splendidly ventilated, and so constructed as to admit of rapid and complete cleansing at the end of each day. An up-to-date operating room is provided in this building for the accommodation of such cases as are ordinarily operated upon in the hospital out-patient service. This operating suite is completely equipped, and has attached to it a ward to be utilized by patients who are convalescing from the minor operations which will be done here.

The equipment of the institution, therefore, makes it one of the most up-to-date of its kind in the city of Philadelphia, and fits it to properly handle its larger number of cases, and render increasingly satisfactory service to its students.

Philadelphia shares the distinction, with three other cities—New York, Chicago and New Orleans—in possessing this institution, the Polyclinic Hospital and School for Graduates in Medicine, conducted solely in the interest of graduate physicians. As such it occupies a field of its own, competing with no other teaching institution in the city, and is the only institution of its kind in Pennsylvania receiving students from every state in the Union, Canada and elsewhere.

It is just such institutions which have given to Philadelphia its present foremost position in the medical world, as the acknowledged center of medical thought, and the home of some of the most progressive and largest medical schools in America.

---

### CLINICAL SOCIETY OF NEW YORK POLYCLINIC MEDICAL SCHOOL AND HOSPITAL. MEETING OF FEBRUARY 5TH, 1912.

A Case of Advanced Carcinoma: Prolongation of Life by Operation.—Presented by Dr. C. A. Frink.

Dr. Frink presented a case of a woman, 48 years of age, widow with three children, one mis-carriage. Her family history showed longevity on both sides. Her husband died of Phthisis 26 years ago. She had one previous attack of appendicitis. Seventeen months ago, patient had attacks of what she called indigestion, with constant pain behind the sternum and vomiting. Never vomited blood, but noticed that food taken several days previously appeared in the vomitus. A diagnosis by herphysician was made of "nervous dyspepsia." She lost weight and strength and the vomiting increased. No lung symptoms were present. On entering the Polyclinic Hospital she could not retain food, and she showed a stenosis of the pyloric valve. She was very

X-ray was unsatisfactory .Stomach contents showed:—presence of Lactic acid, no free Hcl. or Boas bacilli. Urine normal.

Examination of the abdomen showed a mass the size of an orange, situated over the pyloric valve, immovabel. Operation: Rec. 4th, 1908, by Dr. Bainbridge, who did a recto-colic gastro-jejunostomy. The inoperable mass with its enlarged glands which about closed off the pyloris was not touched.

Subsequent History.—June 11th, 1909, the patient returned with her first trouble since operation. Vomiting after food. She otherwise continued in good health, until the spring of '11. In May of this year she showed signs of infection by T. B. and in July the T. B. bacilli were found in the sputum. The patient died of pulmonary T. B. on August 6th, 1911.

Conclusion:—The history of this case emphasizes the importance of operating on cases of cancer that do not appear from the clinical findings to be good surgical risks. This patient was a poor surgical risk, so far as there being any chance of curing her condition by operation. Nevertheless, by doing all in our power, she was able to return to her home and family, enjoying good health, eating and sleeping well, and she gained over 15 lbs. in weight. Two and one-half years after operation she died of a condition entirely independent of her former trouble. We feel that through surgical intervention we prolonged this woman's life, making her a useful member of society for this additional time.

Dr. John A. Wyeth said he considered it the duty of a surgeon to take any and all risks regardless of what might be the result on his reputation or statistics when the patient is in such a state that the conditions seem absolutely hopeless, and death seems imminent, no matter whether the patient dies on the table or not, if in the judgment of the conscientious surgeon, there is a possibility of contributing to the paient's comfort, lessening his discomfort or prolonging his life, it is his duty to undertake the operation. One of the severest criticisms I could make about any man, is that he would not undertake a case if he thought the patient would die.

Dr. Bainbridge said that at the time of operating on this case he had met the conditions exactly as Dr. Weyth had described. The patient had consented, knowing full well her serious condition, and had never ceased to be grateful for her prolonged life. She seemed particularly discouraged in not being able to survive the Phthisis when she had been relieved of the stomach condition which seemed to her to be so much worse.

A Case of Tubercular Peritonitis: Prolongation of Life by Intra-Abdominal Administration of Oxygen.—By Dr. H. D. Meeker.

Dr. Meeker showed a case of tubercular peritonitis which he had treated by the intra-abdominal administration of oxygen. The case was apparently cured. He also advocated its use in cases of profound shock and ascitis, and said that it required from 72 hours to four or five days for complete absorption. Care had to be exercised in watching cases as the abdomen became flat in from 48 to 72 hours. Collapse from its complete absorption should be guarded against, by the free administration of stimulants. Dr. Bainbridge said he had been using oxygen to meet shock in abdominal surgery for the past eight years, and had treated in all about one hundred cases. He had noted marked improvement and a ready response to its use.

A New Pneumatic Electric Proctoscope.—Shown and Demonstrated by Dr. Frank C. Yeomans.

Dr. Yeomans demonstrated a new Proctoscope, and Sigmoidoscope. He said that hitherto the practical methods of directing lighting of protoscopes were by small electric bulbs, carried near the distal ends of the tubes, on insulated carriers, and that they burned out easily. He had adopted the principle of illumination by a more powerful light, within and at the end of the ocular portion of the tube. The Proctoscope is ten inches long, graduated in inches, seven-eights of an inch in diameter, and is fitted with a large flange, at the proximal end of which is perforated by a small tube, joining the main tube at an angle. A light carrier fits very tightly into the auxiliary tube, and a substantial incandescent bulb is covered with a capsule bearing a plano-convex lense, so set that the collected rays are refracted at a compensating angle to the light carrier. This lense only projects into the main tube, and in no practical way interferes with inspection of the bowel, or the passing of instruments.

The ocular end of the tube is enclosed hermetically by a plug which contains a glass window to magnify the illuminated field. A hand bulb attached to a small offset at the side of the plug inflates the bowel to any degree desired. The same light carrier and plug fit the sigmoidoscope which is also fourteen inches long and three-fourths of an inch in diameter.

The points of superiority in this new instrument are:—Simplicity of construction; sterilizable by boiling; excellent illumination by a strong electric lamp which will not burn out readily; and thorough practicability for examination, diagnosis or treatment of lesions in the rectum or sigmoid below its apex.

---

### AUGUSTA MEETING OF THE MEDICAL ASSOCIATION OF GEORGIA.

The recent meeting of the Medical Association of Georgia, held in Augusta, was one of the best of recent years. The registration showed a large number in attendance and the essayists had a good crowd to hear the papers. The hall in which we met was just the right size, so that there was no difficulty in hearing all of the speakers. Most of the time, except on the last day, was devoted to scientific work. The character of the papers was above the usual standard in scientific merit and elicited considerable discussion. The time limit was carefully enforced and thus rapid progress was made and a great variety of subjects were covered.

APRIL 16, 1912.

Meeting of Council, 10.30 p. m. (Eastern time), at Albion Hotel.

WEDNESDAY, APRIL 17—Morning Session.

Meeting called to order at 10.00 a. m. at Moose Club, by President W. L. Fitts, M. D., Carrollton.

Invocation—Rev. Ashby Jones, Augusta.

Address of Welcome on Behalf of City—Hon. Thos. Barrett, Jr., Mayor of Augusta.

Address of Welcome on Behalf of Local Profession—J. M. Hull, M. D., Augusta.

Response—W. F. Westmoreland, M. D., Atlanta.

Report of House of Delegates.

1—"The Local Medical Society and Its Relation to Public Health."

T. J. McArthur, M. D., Cordele.

2—"What the Profession of Georgia has done for Medicine."

T. R. Wright, M. D., Augusta

3—"The Results of Inspection and Examination of Twenty-two Thousand School Children for Diphtheria."

Claude A. Smith, M. D,. Atlanta

4—"The Eugenical Conservation of Man."

A. L. R. Avant, M. D., Savannah

5—"A Clinic with Deaf-Mute Children."

R. C. Woodward, M. D., Adel

6—"Exhibition of X-ray Plates."

John S. Derr, M. D., Atlanta

WEDNESDAY—Afternoon Session.

7—"'Case Histories in Neurology."

Lewis M. Gaines, M. D., Atlanta

8—"So-called Functional Neuroses—Factors Causative and Curative."

Hansell Crenshaw, M. D., Atlanta

9—"Cerebro-Spinal Meningitis."

W. D. Travis, M. D., Covington

10—"Acute Yellow Atrophy of the Liver, with Cases."

T. P. Waring, M. D., Savannah

11—"Tobacco versus Alcohol."

J. G. Dean, M. D., Dawson

12—"Remedial Suggestions—Tenable and Tentative."

R. T. Dorsey, M. D., Atlanta

13—"Flotsam and Jetsam from a Medical Standpoint."

B. P. Oliveros, M. D., Savannah

14—"Cholecystitis as a Complication of Pellagra."

H. F. Harris, M. D., Atlanta

15—"Psychoses Accompanying Pellagra."

E. M. Green, M. D., Milledgeville

16—"Experimental Pellagra."

Geo. C. Mizell, M. D., Atlanta

17—"Salvarsan (606) in Syphilis."

E. G. Ballenger, M. D., Atlanta

18—"Additional Report on the Use of Salvarsan."

W. L. Champion, M. D., Atlanta

19—"Symptoms and Diagnosis of Kidney Diseases."

M. L. Boyd, M. D., Atlanta

20—"The Value of Urethral Catheterization."

W. F. Shallenberger, M. D., Atlanta

21—"Two-step Method of Enucleation of the Prostate."

A. L. Fowler, M. D., Atlanta

22—"Treatment of Pulmonary Tuberculosis by Compression."

S. T. Harris, M. D., Highlands

23—"Treatment of Tuberculosis without the Use of Tuberculin."

J. Monroe Anderson, M. D., Pinedale

24—"The Home Treatment of Tuberculosis."

W. B. Hardman, M. D., Commerce

25—"The Present Status of Specifics in the Treatment of Tuberculosis."

E. C. Thrash, M. D., Atlanta

THURSDAY—Morning Session.

Report of House of Delegates.

26—"Evolution in Aseptic Surgical Technique."

W. F. Westmoreland, M. D., Atlanta

27—"Report of Cases of Gas Bacillus Infection'.'

C. W. Crane, M. D., Augusta

28—"Various Operations for Hernia, with Report of Cases."

L. C. Fischer, M. D., Atlanta

29—"Strangulated Hernia, with Report of Resected Cases."

W. W. Battey, Jr., M. D., Augusta

30—"Ligation of Thyroid Vessels in Exophthalmic Goitre."

W. S. Goldsmith, M. D., Atlanta

31—"The Surgical Treatment of Goitre."

E. G. Jones, M. D., Atlanta

32—"Conservative Treatment of Gunshot Wounds of the Extremities."

Chas. L. Ridley, M. D., Hillsboro

33—Contractions of the Psoas Parvus Muscle Simulating Appendicitis."

G. R. White, M. D., Savannah

34—"Syncytioma Malignum, Surgical Treatment."

Everard A. Wilcox, M. D., Augusta

35—"The Operative Treatment of Recent Fractures."
Frank K. Boland, M. D., Atlanta

36—"The Acute Surgical Abdomen."
Floyd W. McRae, M. D., Atlanta

37—"Intestinal Obstruction, with Report of Experimental Work."
J. L. Campbell, M. D., Atlanta

THURSDAY—Afternoon Session.

38—"Management of Broken Compensation in Cardiac Disorders."
E. E. Murphy, M. D., Augusta

39—"Injurious Effects of Iodides and Bromides on Acute Inflammations, especially Cystitis."
E. Bates Block, M. D., Atlanta

40—"Typhoid Vaccine."
J. W. Palmer, M. D., Ailey

41—"The Rational of Vaccine Therapy."
J. E. Paullin, M. D., Atlanta

42—"Treatment of Typhoid Fever."
S. T. R. Revell, M. D., Louisville

43—"An Unusual Case of Typhoid Fever."
J. E. Summerelfid, M. D., Atlanta

44—"Clinical Typhoid Fever."
W. W. Jarrell, M. D., Thomasville

45—"A Protest Against Unwarranted Reliance Upon Laboratory Findings versus Clinical Evidence in the Diagnosis and Treatment of Disease."
Jas. B. Baird, M. D., Atlanta

46—"Intestinal Sand; Report of Cases in Infants."
N. M. Moore, M. D., Augusta

47—"Clinical Manifestations of Uraemia from a Diagnostic View Point."
Ralston Lattimore, M. D., Savannah

48—"Menstruation Normal and Abnormal."

J. R. B. Branch, M. D., Macon

49—"Parsimony in Nutrition."
Geo. M. Niles, M. D., Atlanta

50—"Amoebic Dysentery, with Report of Cases."
J. M. Sigman, M. D., Savannah

51—"My Observations and Personal Experience on the Improved Technique of Ether Vapor and the Nitrous Oxide-Oxygen Anaesthetics."
T. J. Collier, M. D., Atlanta

52—"Anaesthetic Technique."
Chas. Usher, M. D., Savannah

FRIDAY—Morning Session.

Report of House of Delegates.

53—"The Importance of the Ophthalmoscope in Albuminuric Retinitis of Pregnancy."
Dunbar Roy, M. D., Atlanta

54—"Curettage of the Pharynx in Adults."
M. M. Stapler, M. D., Macon

55—"Extirpation of the Lachrymal Sac in Chronic Dacryocystitis."
F. P. Calhoun, M. D., Atlanta

56—"Malignant Growths of the Pharynx."
J. M. Hull, M. D., Augusta

57—"Some Unusual Cases of Nystagmus."
A. W. Stirling, M. D., Atlanta

58—"Follicular Conjunctivitis: Its Relation to Adenoids."
T. E. Oertel, M. D., Augusta

59—"Nose and Throat Reflexes Causing Cough."
R. R. Daly, M. D., Atlanta

60—"Induction of Labor in Puerperal Eclampsia."

T. J. Carswell, M. D., Waycross

61—"Twin Pregnancy in a Double Uterus."
J. G. Earnest, M. D., Atlanta

62—"Common Errors in Obstetric Practice."
A. J. Kilpatrick, M. D., Augusta

63—"The Interpretation of Surgical Pathology in Upper Right Quadrant."
Willis Jones, M. D., Atlanta

64—"Report of Abdominal Surgical Cases."
G. A. Wilcox, M. D., Augusta

65—"Drainage Sequent to Certain Surgical Procedures."
Jas. N. Ellis, M. D., Atlanta

FRIDAY—Afternoon Session.

Report of House of Delegates.

Election of Officers at 3.00 p. m. Friday.

66—"Cancer Uteri, Return, Treatment, Cure."
E. C. Cartledge, M. D., Atlanta

67—"Oral Surgery."
Robin Adair, M. D., Atlanta

68—"Clinical Phases of Arteriosclerosis."
Thomas D. Coleman, M. D., Augusta
S. H. Visanska, M. D., Atlanta
S. S. Visanska, M. D., Atlanta

70—"Free Dispensary Work."
A. G. Fort, M. D., State Board of Health

81—"The Diagnosis of Positions of the Stomach and Colon by Means of the Roentgen Ray."
C. R. Andrews, M. D., Atlanta

72—"A Resume of the Work of Battle Hill Sanitarium for Tuberculosis of Atlanta."
S. Wickes Merritt, M. D., Alanta

73—"Ulceration of Trigone andVesical Neck a Sequel to Specific Urethritis."
J. L. Farmer, M. D., Savannah

74—"Mental Diseases, Diagnosis and Prognosis."
J. W. Mobley, M. D., Milledgeville

75—"Radical Surgery in Cases of Cancrum-Oris, with Report of Case."
B. S Moore, M. D., Atlanta

76—"The Medical Library and the Medical Society."
V. H. Bassett, M. D., Savannah

77—"A Hernia Case of Historic Interest.
H. M. Michel, M. D., Augusta

78—"Finkelstein Milk—Advantages and Disadvantages in Infant Feeding."
W. A. Mulherin, M. D., Augusta

Entertainment.

## Figures and Tables

**Figure f1:**